# The prevalence of bronchiectasis in patients with alpha-1 antitrypsin deficiency: initial report of EARCO

**DOI:** 10.1186/s13023-023-02830-2

**Published:** 2023-08-12

**Authors:** Robert A. Stockley, Anita Pye, Joshua De Soyza, Alice M. Turner, Marc Miravitlles, the EARCO study investigators, María Torres-Duran, Hanan Tanash, Carlota Rodríguez-García, José Luis López-Campos, Jan Chlumsky, Catarina Guimaraes, Juan Luis Rodríguez-Hermosa, Angelo Corsico, Cristina Martinez-González, José María Hernández-Pérez, Ana Bustamante, David G. Parr, Francisco Casas-Maldonado, Ana Hecimovic, Wim Janssens, Beatriz Lara, Miriam Barrecheguren, Cruz González, Jan Stolk, Christian F. Clarenbach

**Affiliations:** 1 Respiratory Medicine, University Hospitals Birmingham NHS Foundation Trust, Birmingham, UK; 2 Institute of Applied Health Research, University of Birmingham, Birmingham, UK; 3 Pneumology Department, Hospital Universitari Vall d’Hebron, Vall d’Hebron Institut de Recerca (VHIR), Vall d’Hebron Barcelona Hospital Campus, Barcelona, Spain

**Keywords:** Alpha-1 antitrypsin deficiency, Bronchiectasis, Emphysema, Prevalence

## Abstract

**Background:**

Although bronchiectasis has been recognised as a feature of some patients with Alpha1-Antitrypsin deficiency the prevalence and characteristics are not widely known. We wished to determine the prevalence of bronchiectasis and patient characteristics. The first cohort of patients recruited to the EARCO (European Alpha1 Research Collaboration) International Registry data base by the end of 2021 was analysed for radiological evidence of both emphysema and bronchiectasis as well as baseline demographic features.

**Results:**

Of the first 505 patients with the PiZZ genotype entered into the data base 418 (82.8%) had a reported CT scan. There were 77 (18.4%) with a normal scan and 38 (9.1%) with bronchiectasis alone. These 2 groups were predominantly female never smokers and had lung function in the normal range. The remaining 303 (72.5%) ZZ patients all had emphysema on the scan and 113 (27%) had additional evidence of bronchiectasis.

**Conclusions:**

The data indicates the bronchiectasis alone is a feature of 9.1% of patients with the PiZZ genotype of Alpha1-antitrypsin deficiency but although emphysema is the dominant lung pathology bronchiectasis is also present in 27% of emphysema cases and may require a different treatment strategy.

## Background

Alpha-1 antitrypsin (AAT) is a polyvalent protein with many putative functions [1] but thought to be primarily an irreversible inhibitor of neutrophil serine proteinases [2]. These enzymes particularly neutrophil elastase (NE) and proteinase 3 (PR3), have been shown to induce emphysematous and airway changes similar to features of chronic obstructive pulmonary disease (COPD) when instilled into the lungs of experimental animals [2].

Initial studies reported by Eriksson [3] using trypsin inhibitory capacity as the marker of AAT deficiency demonstrated that individuals and family members had clinical features varying from none to severe early onset emphysema and bronchiectasis (Bx). This resulted in widespread testing and reporting of individuals especially those with severe early onset basal panlobular emphysema, sporadic case reports [4] and analysed cohorts [5–8] of those with Bx.

Many patients with COPD have significant bacterial colonisation of the airways and a corresponding neutrophilic load characterised by purulent (elastase positive) secretions [9, 10]. Radiologically these patients often have Bx [10] which lead to the concept that the increased neutrophilic load and hence excess local NE release could damage airways, impair host defences and facilitate bacterial colonisation [11] leading to a self- perpetuating cycle of events [12]. It therefore seems logical that AAT deficiency itself would add another amplifying factor to the pathophysiology of Bx. Indeed in COPD, AAT deficiency is associated with greater airways inflammation (particularly during exacerbations) and detectable active NE even in the stable state [13]; AAT augmentation abrogates this increased inflammation [14].

However, a recent publication has suggested that routine testing of Bx patients for AAT deficiency is not recommended as it is rarely fruitful (< 10%) and does not influence management [15]. This has been questioned [16] as the prevalence of deficiency in such cohorts is clearly higher than expected in the UK indigenous general population [17] and indeed may influence current and future management as systemic AAT augmentation abrogates the airways inflammatory process in AATD [14] and hence excessive putative local airway damage.

The recent establishment of EARCO (European Alpha1 Research Collaboration) International Registry, a deep phenotyping data base of AAT deficiency patients sponsored by the European Respiratory Society (ERS) enables us to estimate the prevalence of Bx in AAT deficiency as a baseline to understanding its nature, impact and management. The current article presents the initial findings of patients recruited to EARCO up to December 2021 [18].

## Methods

### Recruitment

Patients with AAT deficiency were recruited to the ongoing international multicentre observational study to document the natural history of AATD and the impact of augmentation therapy (EARCO study, IRAS ID: 265,728, www.clinicaltrials.gov (ID: NCT04180319)). This clinical research collaboration of the ERS has previously been described in detail by Greulich et al. [19].

Briefly patients over the age of 18 gave written informed consent for their clinical data to be collected during routine assessments at secondary care sites in Belgium, Czech Republic, Croatia, Estonia, Italy, The Netherlands, Poland, Portugal, Romania, Spain, Sweden, Switzerland, Turkey and United Kingdom. All subjects with confirmed AAT deficiency, as defined by serum AAT levels of less than 11microM (50 mg/dl), and/or proteinase inhibitor genotypes ZZ, SZ and heterozygotes or homozygotes for other rare deficient variants were eligible for inclusion in EARCO. Data included extensive baseline demographics as reported recently [19]. For the current study the data base up to December 2021 was searched for all patients with a PiZZ genotype who had undergone a reported CT densitometry scan. Patients were divided into 4 groups namely those with a reported normal scan, evidence of emphysema alone, those with Bx alone and those with reported emphysema and Bx.

### Assessment of CT scans

Bronchiectatic change (namely tubular, cystic or varicose) and the presence of emphysema were reported together with the distribution as either upper or lower zone dominant or widespread. Baseline CT scan data was used to compare prevalence and type of bronchiectasis seen in PiZZ both with and without CT confirmed emphysema.

### Statistical analysis

Patient data for the 4 radiological groups outlined above were analysed using Mann-Whitney U test for FEV_1_, gas transfer and other quantitative variables. Categorical data were analysed using Chi-squared test and p values < 0.05 were accepted as statistically different.

## Results

Data was collected from of the first 860 individual patients recruited to the EARCO registry up to December 2021, of whom 505 had a confirmed PiZZ genotype and 418 (82.8%) of these had a reported CT scan. The baseline characteristics of the whole cohort of PiZZ subjects and the 418 with a CT scan report is shown in Table 1. The majority of patients were never or ex-smokers at the time of recruitment with only 1.2% currently smoking at baseline. Normal scans were described for 77 patients (18.4%). Bx alone was reported on CT scan for 38 PiZZ patients (9.1%) and emphysema alone was reported for 190 patients (45.5%) but was also present with Bx for 113 patients (27%). Overall 303 patients had emphysema reported on their CT scan (72.5%) and of these 39.6% were described as lower zone in distribution, 21.8% upper zone and 38.6% widespread. The bronchiectasis was not characterised in 42% of those with Bx alone or when present with emphysema. It was described as tubular when present alone (36.8%) or with emphysema (36.6%) and cystic (13.2% and 13.6%) alone or with emphysema respectively. When not generalised it was reported as distributed mainly in the lower zones, 43.4% when present alone and 54.5% of those with emphysema, consistent with the archetypal distribution of emphysema in AATD.

**Table 1:** Baseline characteristics of PiZZ patients enrolled in EARCO registry

	Whole cohort	Patients with CT scan
Total, n	505	418
Age, years: mean (SD)	55.26 (13.6)	57.95 (12.2)
Male, n (%)	270 (53.5%)	218 (52.2%)
Smoking, n (%)		
Current	6 (1.2%)	6 (1.4%)
Ex-smoker	290 (57.4%)	249 (59.6%)
Never	208 (41.2%)	162 (38.8%)
Unknown	1 (0.2%)	1(0.2%)
Pack years: Mean (SD)	18.70 (13.75)	18.77 (13.80)
Post-bronchodilator FEV_1_%: Mean (SD)	62.56% (29.54)	61.42% (29.14)
Kco %: Mean (SD)	67.95% (22.91)	66.37% (22.92)

Data is shown for the whole cohort and those with a reported CT scan. Data is summarised as Mean and standard deviation in parentheses as indicated. All other data is number and percentage of each cohort. FEV_1_ is post bronchodilator where available and gas transfer (Kco) is reported as the transfer coefficient corrected for alveolar ventilation

Of the 87 patients in the PiZZ group who had no CT scan report the demographics were similar to the group as a whole with 59.8% male a mean age of 47.8 years (SD = 16.0). Most (52.9%) were never smokers and 47.1% ex-smokers with a mean pack year history of 16.0 (SD = 16.5). Of these 87 patients the average FEV_1_ was 88.3% (SD 31.2). Kco was available for 54 of the 87 patients with a mean value of 76.7% predicted (SD 20.9). The baseline characteristics of those with CT scan reports were similar to the total PiZZ cohort (Table 1).

**Fig. 1:**
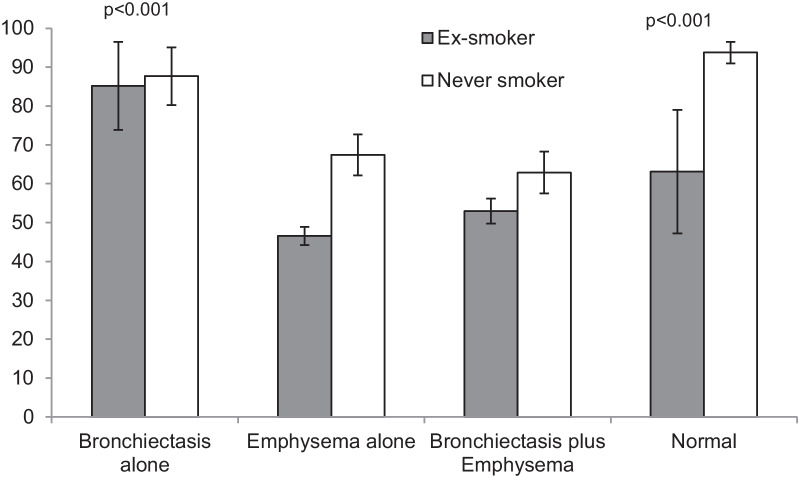
Post-bronchodilator FEV_1_% predicted in patients with PiZZ alpha-1 antitrypsin deficiency according to CT pathology. Histograms are mean data for ex-smokers and never smokers with SD bars. p values indicate differences compared to emphysema alone and the combined group

**Fig. 2:**
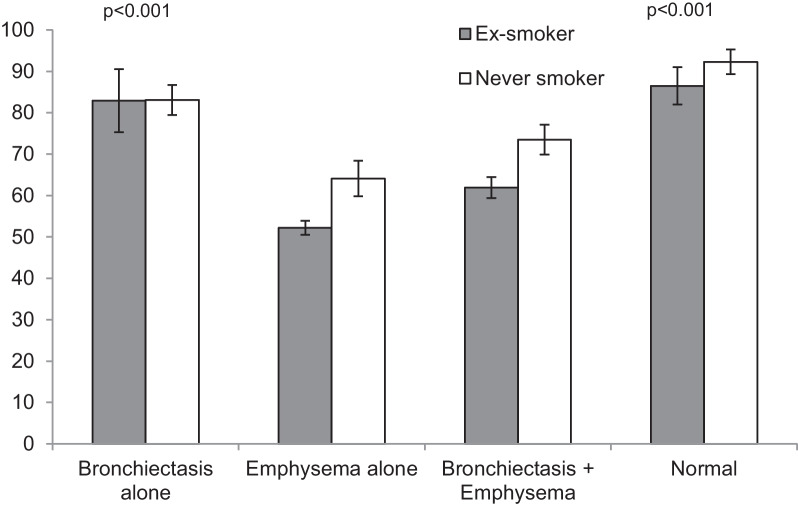
Kco % predicted in patients with PiZZ alpha-1 antitrypsin deficiency according to CT pathology. Histograms are the mean with SD bars for ex-smokers and never smokers p values indicate differences compared to emphysema alone and the combined group

The four groups of PiZZ subjects defined by the CT findings are summarised in Table 2. There were some demographic differences between the 4 groups. Those with a normal scan were predominantly female, mainly never smokers and younger with generally near normal lung function compared to the groups with emphysema (p < 0.001). The demographics for this “normal” group was similar to the group of patients who had Bx alone (Table 2). The patients with emphysema were older on average (p < 0.001) with a slight male preponderance (p = 0.09), consisted of fewer never smokers and had a greater smoking history compared to those with a normal scan or Bx alone (p < 0.001). In addition, these 2 emphysema groups (with and without Bx) had moderate airflow obstruction and significantly reduced gas transfer as expected (p < 0.001 compared to those with normal scans or Bx alone). The patients with emphysema and Bx were more likely to be male (p = 0.021), smokers (p < 0.001) and with reduced lung function compared to those with Bx alone (p < 0.001 all measures). The average lung function data corrected for age sex and height for the 4 groups is summarised in Figs. 1 and 2.

**Table 2:** Baseline characteristics of 418 PiZZ patients split according to CT scan findings

	Bronchiectasis alone	Emphysema alone	Bronchiectasis plus Emphysema	Normal CT scan
Total, n	38	190	113	77
Age, years: Mean (SD)	57.2 (10.2)	58.8 (9.9)	61.1 (11.0)	46.2 (14.0)
Male, n (%)	11 (28.9%)	112 (58.9%)	59 (52.2%)	36 (46.8%)
Smoking, n (%)				
Current	0	4 (2.1%)	0	2 (2.6%)
Ex-smoker	12 (31.6%)	140 (73.7%)	77 (68.1%)	20 (26.0%)
Never	26 (68.4%)	46 (24.2%)	36 (31.9%)	54 (70.1%)
Unknown	0	0	0	1 (1.3%)
Pack years: Mean (SD)	14.0 (15.7)	20.8 (13.6)	17.1 (10.1)	9.35 (8.8)
Mean FEV_1_% Post BD (SD)	98.3% (18.0)	56.2% (24.3)	59.6% (27.5)	92.6% (20.2)
Mean Kco % (SD)	83.1% (17.8)	54.7% (18.9)	65.1% (19.1)	90% (16.9)

### Prevalence in patients with the SZ genotype

The milder SZ genotype is not considered an at risk variant for COPD in the absence of heavy smoking. However similar data was documented within the EARCO database for a number of such patients (n = 235) of whom 62.1% also had a CT scan. Of these 41.8% were reported as normal, 11.6% had bronchiectatic changes alone, 32.2% emphysema alone and 14.4% both Bx and Emphysema. These patients had a higher smoking history than the ZZ cohort (overall 30.55 pack years; SD 27.6) and predominantly normal spirometry (see Table 3).

**Table 3:** Baseline characteristics of 146 PiSZ patients split according to CT scan findings

CT scan performed (% of pts)	146 (62.1%)	Pack years	FEV1% predicted Pre-bronchodilator	KCO % predicted
Bx alone Current smoker Ex-smoker Never	17 (11.6%) 2 (11.8%) 6 (35.3%) 9 (52.9%)	ND 16.5 (14.0%) 0	ND 104.3 (22.7) n = 5 89.7 (14.4) n = 7	ND 96.1 (13.3) n = 6 93.1 (11.4) n = 7
Emphysema alone Current smoker Ex-smoker Never	47 (32.2%) 10 (21.2%) 31 (66.0%) 6 (12.8%)	32.6 (23.7) 41.3 (24.7) 0	95.1 (21.1) n = 9 88.2 (21.2) n = 22 ND	72.7 (20.3) n = 8 64.7 (23.5) n = 26 72.4 (31.5) n = 5
Emphysema + Bx Current smoker Ex-smoker Never	21 (14.4%) 3 (14.2%) 17 (81.0%) 1 (4.8%)	ND 38.2 (36.7) 0	ND 76.8 (34.5) n = 12 ND	ND 68.5 (20.3) n = 15 ND
Normal CT scan Current smoker Ex-smoker Never	61 (41.8%) 2 20 39	ND 12.1 (10.4) 0	ND 94.1 (20.2) n = 15 101.6 (18.6) n = 29	ND 99.5 (17.0) n = 15 90.7 (13.8) n = 30

Data is shown as number and % in parentheses or mean and SD

## Discussion

The baseline data from this initial analysis of the first 860 patients uploaded to the EARCO data base identified 505 PiZZ patients of whom 418 had undergone CT scanning as part of their disease characterisation and of these 38 had evidence of Bx alone which is similar to the prevalence found by Eriksson [3] in his initial 23 patient cohort analysis. Interestingly the small sub cohort reported here was predominantly female and with little smoking history (which may partly explain the lack of emphysema) and predominantly normal lung function. This sex and smoking difference is reminiscent of historical studies where smoking men with the same symptoms but airflow obstruction were often assumed to have COPD and investigated no further, whereas non-smoking females underwent more extensive investigations including radiographic bronchograms. It was often mooted that this reflected a selection bias dependant on investigation whereas here that bias seems unlikely.

Clearly Bx alone is a feature of a proportion of patients with PiZZ AATD and is similar to the prevalence described by Carreto et al. in specialist Bx clinics [15] which suggests that continued testing in Bx for this associated genetic defect should still be continued. Whether this leads to different management to non-deficient Bx patients requires much more individual characterisation including bacterial colonisation, airways neutrophilia (and serine proteinase activity), as well as exacerbation history. Whereas this is important in all patients with exacerbations the presence of AATD will likely have an increased inflammatory load [12] amenable (at least in part) to AAT augmentation intravenously [13], by the inhaled route [20] or with more recent oral antiproteinase strategies in development [21].

However with the advent of CT scanning as a non-invasive GOLD standard more cases of Bx are being identified with up to 30% of COPD patients having both emphysema and Bx [22] suggesting it is a significant comorbidity especially in severity subgroups [23]. In the current study 113 (27%) of PiZZ patients had Bx associated with emphysema which is also similar to the prevalence of easily visible changes described by Parr et al. in a smaller but highly characterised cohort [15], although the prevalence of minor changes that fulfilled the Naidich criteria for Bx [24] was almost universal. In non-deficient COPD this association influences both mortality [25] and recurrent exacerbations [26]. Indeed early studies of COPD patients with productive purulent sputum suggesting significant bacterial colonisation [9] is associated with a high prevalence of Bx [10]. On this basis it has been suggested that such COPD patients with Bx should be considered for additional treatment strategies as for patients with Bx alone [22] Whether these features are also true of a significant proportion of AATD patients remains to be determined but it is tempting to speculate that (as in COPD) such patients have increased inflammation and may benefit from antiproteinase therapy and specifically Cathepsin C inhibitors [27] that may reduce both the inflammation and proteinase load in Bx but also influence the proteinase dependant emphysema in AATD patients.

The increasing recognition of the associations of Bx with COPD has raised the concept that this represents a treatable trait and the arguments above provide a strategy in both non deficient and AAT deficient patients. More recently a workshop has described the ROSE criteria [28] for the key components to determine the implications of the combination and its verification. As this recent work was not published when EARCO was established the essential components of the ROSE score were not mandatory in the data base and much of the features of ROSE have not been systematically collected to date. Also, COPD related to AATD (although highly susceptible to smoking which is a key component of the ROSE score) can also develop in never smokers and hence a “ROSE score” may require further adjustment for this component. Clearly this association and score needs further exploration in AATD as well as non-deficient COPD as mentioned by the authors [28].

The current study has the strengths of being an in depth characterisation of AAT deficient patients across many specialist groups and countries and is therefore reflective of the current patient population. It emphasises that many patients do not fulfil the archetypical AATD patient with basal panlobular emphysema, supporting widespread AAT testing in all patients with COPD and those who present with Bx alone. However it does have some weaknesses. In particular, not all patients had CT scans at baseline (although those who did not, had similar baseline characteristics of those who did). In addition data on airway colonisation, and nature and frequency of exacerbations are not mandatory fields on the EARCO data base, leading to missing data. The nature and distribution of emphysema as well as characteristics of the Bx are requested within EARCO, but again not mandatory, and should also be described to strict criteria as part of subsequent retrospective and prospective analyses to complete understanding of this combination phenotype and its management. Finally, tests to confirm that other causes of Bx had been excluded were rarely formally reported in the EARCO database, although sites confirmed that it was routine practice to screen for immunodeficiency, take a detailed history of past infections and consider autoimmune or other familial causes.

The current retrospective analysis has concentrated on those with the recognised “at risk” PiZZ genotype. Although EARCO includes other rarer severe deficiency genotypes the numbers are currently too few for meaningful comparisons with the core ZZ cohort.

The milder SZ genotype is not considered “at risk” for COPD in the absence of heavy smoking [29] and currently not recommended for augmentation therapy. In the EARCO data base of the 235 SZ patients documented the cohort had a higher smoking history but a greater proportion of normal scans. and predominantly normal spirometry. Bronchiectasis alone and Emphysema with and without bronchiectasis was also observed.

The reasons for testing these patients for AAT genotype (although permitted for the EARCO data base) is currently unknown but it is clearly important to determine any influence of acquisition bias in a prospective study to explore the nature and impact of these findings in the SZ genotype.

## Conclusions

In conclusion AAT deficiency is associated with Bx alone in up to 10% of PiZZ individuals and 27% of those with emphysema. This should become a routine part of patients’ assessment and become a feature of in depth future clinical study and management. In particular this should include microbiology the airways inflammation compared to non-deficient COPD as well as the impact on progression and health status and the need for, and effect of antiproteinase therapy.

## Data Availability

The datasets generated and/or analysed during the current study are not publicly available due to individual patient privacy and lack of consent but are available from the corresponding author on reasonable request.

## Abbreviations

AAT: Alpha-1 antitrypsin
NE: Neutrophil elastase
PR3: Proteinase 3
COPD: Chronic obstructive pulmonary disease
Bx: Bronchiectasis
AATD: Alpha-1 antitrypsin deficiency
EARCO: European Alpha1 Research Collaboration) International Registry
ERS: European Respiratory Society
